# When advisors do not know what is best for advisees: Uncertainty inhibits advice giving

**DOI:** 10.1002/pchj.745

**Published:** 2024-03-26

**Authors:** Ruida Zhu, Honghong Tang, Jinghua Xue, Yuanping Li, Zilu Liang, Simeng Wu, Song Su, Chao Liu

**Affiliations:** ^1^ Department of Psychology Sun Yat‐Sen University Guangzhou China; ^2^ Business School Beijing Normal University Beijing China; ^3^ State Key Laboratory of Cognitive Neuroscience and Learning & IDG/McGovern Institute for Brain Research Beijing Normal University Beijing China; ^4^ Center for Collaboration and Innovation in Brain and Learning Sciences Beijing Normal University Beijing China; ^5^ Beijing Key Laboratory of Brain Imaging and Connectomics Beijing Normal University Beijing China

**Keywords:** advice giving, motivation to influence, sense of power, uncertainty, worry about harm to others

## Abstract

While seeking advice can be beneficial for advisees, advisors may not always possess the necessary knowledge to provide appropriate guidance. Poor‐quality advice can mislead advisees rather than offering assistance. Despite the research interest in advisees, few studies have investigated advisors' psychological and behavioral responses, especially when they faced uncertainty regarding the optimal course of action for advisees. To fill this gap, we developed novel paradigms aiming at manipulating advisors' uncertainty, allowing for a systematic investigation of advisors' behavior, motivation, and emotion. Across four studies, we consistently found that advisors under uncertainty give less advice. Furthermore, we observed that uncertainty modulates advisors' motivation to influence, worry about harm to others, and/or sense of power. The motivation to influence and/or worry about harm to others can mediate the effect of uncertainty on advice giving. Besides, we identified nuanced distinctions in the effects of ambiguity and risk, two distinct types of uncertainty, on advisors' psychological processes. Our findings shed light on advisors' self‐monitoring of the quality of their advice, thereby contributing to a deeper understanding of advisor–advisee communication from the perspective of advisors.

## INTRODUCTION

Limited information often renders individuals uncertain about the most suitable course of action in unfamiliar situations. Seeking advice from others, commonly known as advisors, offers a convenient means to mitigate this uncertainty (Einhorn & Hogarth, 1975; Mirc & Parker, 2020). Extensive research has demonstrated that individuals engage in evaluating the quality of advice and the characteristics of advisors (Bonaccio & Dalal, 2006; Gaertig & Simmons, 2018; Haran & Shalvi, 2020; Leong & Zaki, 2018; Zhang & North, 2020), and can effectively reduce uncertainty in the communication with advisors who possess necessary information (Jonas et al., 2005; Sniezek & Van Swol, 2001). Nevertheless, it is not rare for advisors themselves to face uncertainty regarding the most optimal choices for advisees. In such cases, advisors' responses matter, as misguided advice resulting from a lack of information can lead advisees astray rather than offering genuine assistance (Heath & Gonzalez, 1995; Isopi et al., 2014). Despite the substantial research interest in advisees' responses to uncertainty in the advisor–advisee communication (see reviews, Bonaccio & Dalal, 2006; Kämmer et al., 2023; Rader et al., 2017), the effect of uncertainty on advisors is largely unknown.

Advisors' and advisees' concerns and emotional experiences are asymmetric. Advisors want to exert influence on advisees and may feel offended when advisees seek advice from multiple advisors, whereas advisees prefer to seek advice from multiple advisors to acquire a comprehensive range of information (Blunden et al., 2019). Advisors derive a sense of power from their role in providing advice (Schaerer et al., 2018), while advisees may experience a sense of stigma when receiving advice (Eskreis‐Winkler et al., 2018; Fisher et al., 1982). This asymmetry suggests that the effect of uncertainty on advisors may be distinct from that on advisees. An examination from the perspective of advisors is needed.

Uncertainty has potential to impact advice giving in three possible ways. Advice giving is regarded as a form of social influence through which individuals aim to shape others' behavior to achieve personal goals (Guntzviller et al., 2020; Peluso et al., 2017; Rader et al., 2017). Prior studies have revealed that advisors give advice with the intent of helping advisees for altruistic purposes (Goldsmith & Fitch, 1997; Hennig‐Thurau et al., 2021) or fulfilling self‐interest for selfish motives (Barneron & Yaniv, 2020; Cain et al., 2005; Sah & Loewenstein, 2012). For example, in the context of healthcare, a medical advisor with altruistic purposes would recommend treatment plan A over B if they possess clear knowledge that plan A is more beneficial for the patient. However, when advisors confront uncertainty regarding the outcomes of advisees' choices, their ability to achieve their goals diminishes (Ove Hansson, 1996). For instance, if a medical advisor is uncertain about whether treatment plan A is superior to plan B for the patient, their capacity to assist the patient through high‐quality advice is compromised, potentially leading to a reduced inclination to influence the patient's decision through advice giving. Hence, it is possible that uncertainty diminishes advice giving by attenuating advisors' motivation to influence advisees.

Advice giving is an interpersonal behavior that can cause various interpersonal consequences. Inadequate advice from advisors with limited knowledge reduces advisees' choice accuracy and harms advisees' benefits (Heath & Gonzalez, 1995; Isopi et al., 2014). Advisors who give advice are supposed to take responsibility for advisees' failures (Bonaccio & Dalal, 2006; Harvey & Fischer, 1997). Besides, giving advice is regarded as a signal of advisors' willingness to provide emotional support (Goldsmith & Fitch, 1997; MacGeorge et al., 2004). Withholding advice may result in advisors being considered to be indifferent toward advisees and incur negative evaluations from others (Rader et al., 2017). Previous studies have found that advisors have concern about the interpersonal consequences of their advice (Barneron & Yaniv, 2020; Mahmoodi et al., 2018). Notably, uncertainty may affect advisors' interpersonal concerns. Under uncertainty, advisors may perceive a heightened risk of assuming responsibility for causing harm to advisees (e.g., Dana & Cain, 2015) and find justification for withholding advice by emphasizing their lack of knowledge rather than an indifference toward the advisee's situation. Therefore, another possibility is that uncertainty dissuades advisors from giving advice through changing their interpersonal concerns (e.g., worry about harm to others, responsibility, or worry about evaluation from others).

Advice giving has a close association with a sense of power (Peluso et al., 2017; Schaerer et al., 2018). Compared to receiving advice, individuals tend to perceive a greater sense of power when they give advice (Schaerer et al., 2018). It is suggested that advice giving is facilitated by a sense of power. The sense of power stems from two primary sources: subjective feelings of control over others' behaviors (e.g., whether to invest in a company) and control over others' outcomes (e.g., whether the investment will yield a profit; Anderson et al., 2012; Schaerer et al., 2018). Both sources are related to uncertainty. In situations where advisors are uncertain about the potential outcomes associated with each of the advisee's options (e.g., lacking knowledge regarding the profitability of an investment), they may expect their advice to be rejected due to its perceived low quality, leading to a diminished sense of control over others' behaviors. Moreover, even if advisees were to accept the advice (e.g., deciding to invest), advisors who possess no insight into the consequences of the decision (e.g., the profitability of the investment) would still lack a sense of control over others' outcomes. Consequently, uncertainty may reduce advisors' sense of power. Thus, an additional possibility emerges. Uncertainty inhibits advice giving via decreasing advisors' sense of power.

Studying the effect of uncertainty on advisors' sense of power not only is crucial to understanding advisors' psychological activities, but also may extend the theories on a sense of power. Previous studies have predominantly focused on investigating various consequences of a sense of power (for reviews, see Anderson & Brion, 2014; Magee & Galinsky, 2008; Tost, 2015), but how and when a sense of power comes about are relatively unexplored (Schaerer et al., 2018). Though real power in life, such as holding a management position, and interpersonal influence attempts, like advice giving, have been identified as contributors to the sense of power, it is important to identify new factors that influence sense of power (Schaerer et al., 2018; Tost, 2015). Uncertainty may be one such influential factor.

Uncertainty can be further divided into two different psychological constructs: risk and ambiguity (Raiffa, 1961). Under risk, people know the probabilities of possible outcomes; under ambiguity, the probabilities of possible outcomes are (partly) unknown. Given their different psychological features (Blankenstein et al., 2021; Sherman, 1974), risk and ambiguity may have distinct effects on advisors' psychological processes. It has been documented that people are more averse to ambiguity compared to risk (Camerer & Weber, 1992; Curley et al., 1986; Slovic & Tversky, 1974). The stronger negative feelings about ambiguity, relative to risk, can result in larger effect on attenuating advisors' positive feelings, such as their sense of power (Russell, 2003). Additionally, events under ambiguity, due to the lack of information regarding probabilities, are supposed to be more unpredictable than those under risk (Hsu et al., 2005; Jenkins & Mitchell, 2010; Volz et al., 2004). This heightened unpredictability associated with ambiguity may elicit greater interpersonal concerns (e.g., worry about harm to others). Consequently, it is necessary to examine how risk and ambiguity respectively contribute to advisors' psychological activities (Blankenstein et al., 2021).

In this research, we conducted four studies to examine the effect of uncertainty on advice giving. Based on the theoretical reasoning above, we hypothesized that uncertainty would inhibit advice giving. We also tested whether uncertainty affects advisors' motivation to influence, interpersonal concerns, and/or sense of power and whether they mediate the relationship between uncertainty and advice giving (potential psychological mechanisms of uncertainty‐modulated advice giving; Agler & De Boeck, 2017). As this research field has not been extensively studied, there is no evidence to suggest that one mechanism ought to be dominant over others. The mediation analyses were performed for exploratory purposes (Losin et al., 2020). We developed novel paradigms to manipulate advisors' uncertainty. In Studies 1 and 2, we employed a combined manipulation of ambiguity and risk, whereas Study 3 focused on pure ambiguity, and Study 4 examined pure risk. We report how we determined our sample sizes, all data exclusions, all manipulations, and all measures in the studies.

## STUDY 1

### Methods

#### Participants and design

How we determined the sample size and more data description for all studies are illustrated in the Supplementary information (SI) (SI 1 and 2). One hundred and forty‐eight college students participated in our experiment. Invalid responses from 14 participants were excluded (see SI 3), leaving 134 participants in the analyses (119 females, 15 males, *M*_age_ = 18.75 years, *SD*_age_ = 0.78 years; 67 in the uncertainty condition, 67 in the certainty condition). Our study had a between‐subject design.

#### Procedure and measures

At the beginning, participants completed a power scale by rating how strongly they had various types of feelings (7‐point scale; 1 = *not at all*, 7 = *a great deal*). In this scale, four items were included for measuring a sense of power (“powerful,” “in control,” “strong,” and “influential”; Power 1, power baseline, Cronbach's *α* = .76; Schaerer et al., 2015), and the others were unrelated fillers that disguised the purpose of the measurement (“nervous,” “anxious,” “happy,” “exhausted,” and “confused”; Schaerer et al., 2018). Then, the participants were required to recall and write down an event. In the uncertainty condition, the instructions read:

*Please recall and write down a recent event in which someone sought advice from you. In this event, you were uncertain about what to do was better for the questioner*.

In the certainty condition, the instructions read:

*Please recall and write down a recent event in which someone sought advice from you. In this event, you were certain about what to do was better for the questioner*.

After the recall, the participants indicated the extent to which they were certain about what to do was better for the questioner in the event (7‐point scale; 1 = *completely uncertain*, 7 = *completely certain*; certainty rating) and how long ago the event happened (unit: week) and then completed the power scale for the second time (Power 2; Cronbach's *α* = .90). Afterwards, the participants were asked to recall and write down how they responded to the questioner in the event. Following the recall, the participants indicated which of the two statements could better describe their response (0 = *I did not give clear advice [kept advice]*, 1 = *I gave clear advice [gave advice]*), rated how clear their advice was (7‐point scale; 1 = *did not give clear advice*, 7 = *gave very clear advice*), and completed the power scale for the third time (Power 3, Cronbach's *α* = .92). Afterwards, they also answered several questions about their motivations on a 7‐point scale (1 = *not at all*, 7 = *very much*): “To what extent did you want to influence the questioner's decision?” (motivation to influence; one item); “To what extent did you want to harm the questioner?” (motivation to harm, one item; this helped us to know whether the influence participants wanted to exert on the questioner was malicious); “To what extent did you worry that your advice might harm the questioner's benefits if you gave advice?” (worry about harm to others; one item); “To what extent did you worry that you might leave a bad impression on the questioner if you did not give advice?” (worry about evaluation from others; one item); and “To what extent did you take responsibility for the advice if you gave advice?” (responsibility; one item). An attention check was embedded in the questions (SI 4).

A recent study on the effect of advice giving on sense of power measured participants' sense of power at two time points (i.e., one before participants knew the situation related to advice [i.e., Power 1] and one after participants completed the advice behavior [i.e., Power 3]; Schaerer et al., 2018). Besides those two time points, we measured an additional one when advisors were aware of the situation related to advice giving but before they engaged in advice behavior (i.e., Power 2). These measures, on one hand, allow us to test whether we can replicate previous findings (about Power 1 and 3; Schaerer et al., 2018); on the other hand, they enable us to examine whether a sense of power (especially Power 2) mediates the effect of uncertainty on advice giving.

### Results and discussion

#### Manipulation check

**FIGURE 1:**
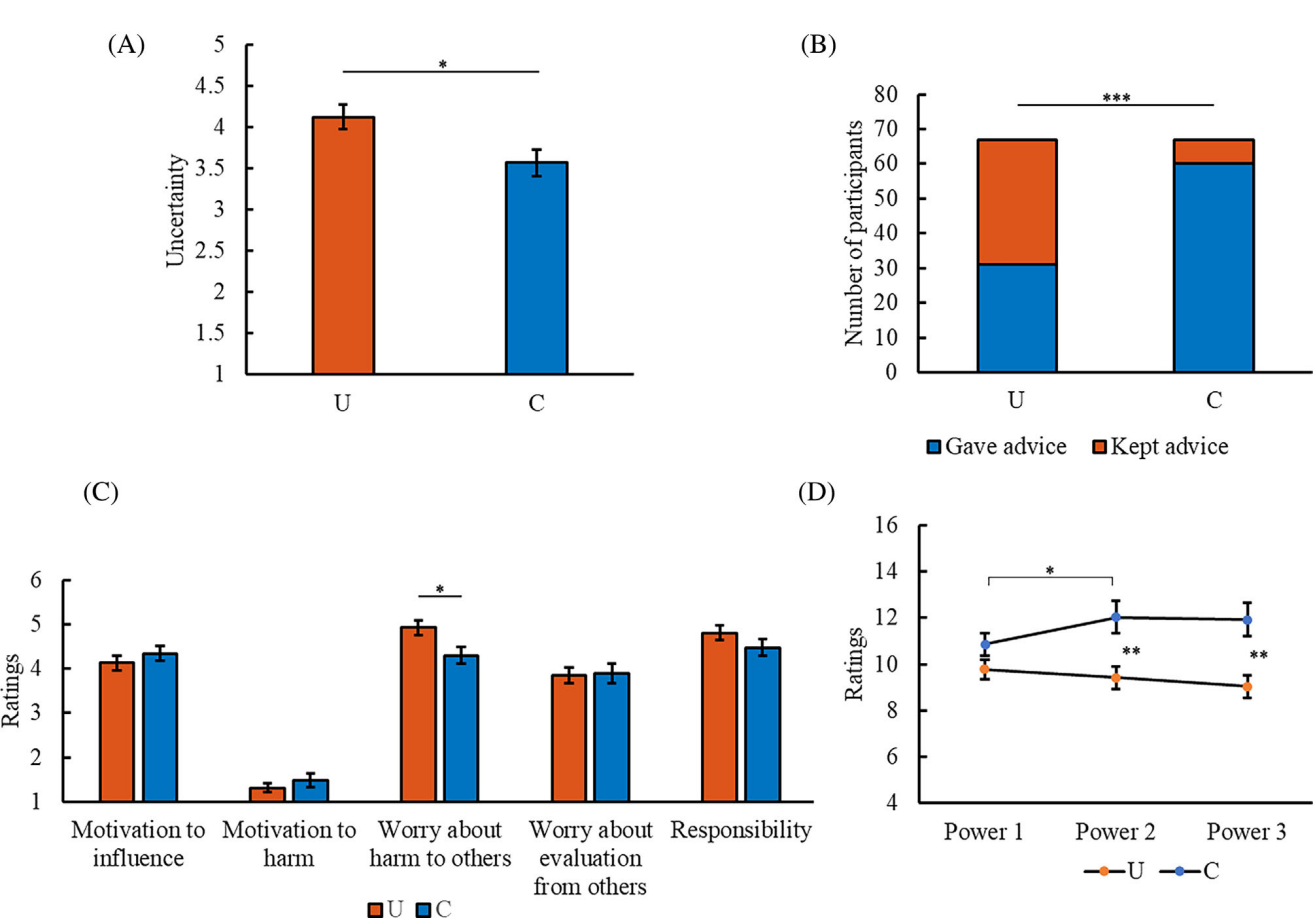
Results of Study 1. (A) Mean uncertainty ratings (± *SE*) in the uncertainty (U) and certainty (C) conditions. (B) Number of participants who gave advice to others or kept advice to themselves in the U and C conditions. (C) Mean ratings of motivations related to advice giving (± *SE*) in the U and C conditions. (D) Mean ratings of sense of power at different time points (± *SE*) in the U and C conditions. *p < .05, **p < .01, ***p < .001.

For ease of understanding, ratings of certainty were reverse coded into ratings of uncertainty (i.e., 1–7, 2–6 and so on). We reported the uncertainty ratings across all studies. The participants in the uncertainty condition were more uncertain than those in the certainty condition (*F*(1,124) = 6.47, *p* = .012, partial *η* ^2^ = .050; Figure 1A), which indicates a successful manipulation of uncertainty.

No significant difference was found in the occurrence time of recalled events between the uncertainty (*M* = 2.69, *SD* = 3.16) and certainty (*M* = 2.33, *SD* = 1.87) conditions (*F*(1,117) = 0.61, *p* = .438, partial *η* ^2^ = .005). The occurrence time of recalled events was unlikely to explain any significant differences we might observe between the conditions.

#### Advice giving

The percentage of participants who gave advice or kept advice differed between the conditions (*χ* ^2^(1,134) = 28.80, *p* < .001, Cramer's *V* = .464; Figure 1B). The participants in the uncertainty condition (*M* = 3.72, *SD* = 1.46) gave less clear advice than those in the certainty condition (*M* = 5.46, *SD* = 1.56; *F*(1,122) = 40.85, *p* < .001, partial *η* ^2^ = .251). The results support that uncertainty decreases advice giving.

#### Motivations related to advice giving

The participants in the uncertainty condition had more worry about harm to others (*F*(1,132) = 6.33, *p* = .013, partial *η* ^2^ = .046) than those in the certainty condition (Figure 1C). There was no significant difference in the motivation to influence, motivation to harm, worry about evaluation from others, or responsibility between the conditions (all *Fs*< 1.77, *ps*< .186, partial *η* ^2^s< .013).

No significant correlation between the motivation to influence and motivation to harm was found (Pearson correlation *r* = .04, *p* = .612, *N* = 134). No evidence shows that the influence participants wanted to exert on the questioner was malicious.

#### Sense of power

No significant difference was found in Power 1 between the conditions (*F*(1,132) = 2.91, *p* = .091, partial *η* ^2^ = .022), which indicates that the participants in different conditions had a similar level of power baseline. Replicating the previous findings (Schaerer et al., 2018), we found that compared to keeping advice, giving advice increased the sense of power (Power 3; *F*(1,132) = 3.92, *p* = .050, partial *η* ^2^ = .029).

Then, we focused on examining the effect of uncertainty on the sense of power. The participants in the uncertainty condition had lower Power 2 (*F*(1,132) = 9.35, *p* = .003, partial *η* ^2^ = .066) and Power 3 (*F*(1,132) = 10.99, *p* = .001, partial *η* ^2^ = .077) than those in the certainty condition (Figure 1D). To test the robustness of the findings, we also (1) conducted analyses of covariance involving Power 1 and advice giving as covariates and (2) examined the effect of uncertainty on sense of power when uncertainty was indexed by uncertainty ratings instead of conditions (uncertainty vs. certainty). Power 1 and advice giving were entered as covariates, as a study found that they had significant effects on the sense of power (Schaerer et al., 2018). Overall, the results confirm the significant effect of uncertainty on the sense of power (in Studies 1, 2, and 3) (SI 5).

Besides, we examined the dynamic changes in the sense of power in the uncertainty and certainty conditions. In the uncertainty condition, Power 2 was not different from Power 1 (*F*(1,66) = .86, *p* = .358, partial *η* ^2^ = .013); Power 3 was not different from Power 1 (*F*(1,66) = 3.08, *p* = .084, partial *η* ^2^ = .045) or Power 2 (*F*(1,66) = 1.24, *p =* .269, partial *η* ^2^ = .018). In the certainty condition, Power 2 was higher than Power 1 (*F*(1,66) = 4.38, *p =* .040, partial *η* ^2^ = .062); Power 3 was not different from Power 1 (*F*(1,66) = 3.18, *p = *.079, partial *η* ^2^ = .046) or Power 2 (*F*(1,66) = .12, *p =* .730, partial *η* ^2^ = .002).

The findings suggest that recalling an uncertain situation related to advice (without recalling advice behavior) is sufficient to inhibit advisors' sense of power.

#### Mediation effect

We tested for psychological mediators of the uncertainty‐modulated difference in advice giving using a two‐stage process (Losin et al., 2020). In the first stage, we searched for significant differences between the uncertainty and certainty conditions in psychological factors that may influence advice giving. Power 1 and Power 3 were not on the search list, as they were not affected by our uncertainty manipulation (Power 1) or occurred after the advice behavior (Power 3). The identified psychological factors were considered as candidate mediators. In the second stage, we tested whether any of the candidate mediators mediated the effect of uncertainty on advice giving using the PROCESS macro based on SPSS software. Data from two conditions were combined. We examined the mediation effect, with manipulation of uncertainty (1 = uncertainty condition, 0 = certainty condition) as the predictor variable (*X*), advice giving (0 = kept advice, 1 = gave advice) as the outcome variable (*Y*), and ratings on candidate psychological mediators as the mediator variable (*M*; one candidate mediator per mediation analysis). We used a bootstrap procedure (5000 samples) to obtain 95% confidence intervals (CIs) of path coefficients for significance testing. An effect was considered as significant when the CI of a path coefficient did not cover zero.

Worry about harm to others and Power 2, which revealed significant differences between the conditions, were selected as candidate mediators. However, none of them revealed a significant mediation effect (Table S6). For completeness, we also tested whether any other psychological factor had a mediation effect. None of the other mediation effects reached significance.

Based on the suggestion of an anonymous reviewer, we also used a serial multiple mediation analysis to test the possibility that an indirect effect of uncertainty on advice giving is achieved through the worry about harm to others and motivation to influence in sequence (i.e., uncertainty → worry about harm to others → motivation to influence → advice giving). The indirect effect of uncertainty on advice giving through worry about harm to others tendency and harm avoidance in sequence was not significant (see SI 6).

Failing to find any significant mediation effect may be because of the recall paradigm we used. Although recall paradigms are commonly adopted by studies on advice, recall bias and difference in the content of recall memory might confound the mediation effects to some extent. Clearly reporting these insignificant mediation effects here helps future studies to select appropriate paradigms. In Study 2, we circumvented the potential problems of Study 1 by using an imagination paradigm.

## STUDY 2

### Methods

#### Participants and design

One hundred and fifty‐eight college students participated in our experiment. Invalid responses from 11 participants were excluded (SI 3), leaving 147 participants in the analyses (78 females, 69 males, *M*_age_ = 19.47 years, *SD*_age_ = 1.00 years; 72 in the uncertainty condition, 75 in the certainty condition). Our study had a between‐subject design.

#### Procedure and measures

Participants completed a power scale at the beginning (Power 1, Cronbach's *α* = .80; Schaerer et al., 2018). Then the participants imagined that they were in the following scenario:

*Your friend planned to educate him/herself with some knowledge related to your major. Two textbooks (textbook A and textbook B) might be suitable for him/her, which were of a price. Your friend could afford only one of them. Therefore, your friend sought advice from you*.

In the uncertainty condition, the participants imagined:

*As you would not take any related courses until the next semester, you were uncertain which textbook was better. You only vaguely remembered that someone said textbook A was better than textbook B*.

In the certainty condition, the participants imagined:

*As you took related courses this semester, you were certain that textbook A was better than textbook B in all aspects*.

After imagining the scenario, the participants indicated which textbook was better based on their opinions (textbook A or textbook B), indicated the extent to which they were certain about which textbook was better (1 = *completely uncertain*, 7 = *completely certain*) and completed the power scale for the second time (Power 2, Cronbach's *α* = .91). Afterwards, the participants imagined how they would respond to their friends. The participants were asked whether they wanted to give advice to the friend or kept the advice to themselves (i.e., telling the friend they did not know; 0 = *kept advice*, 1 = *gave advice*). For the participants who decided to give advice, they were asked to report what advice they wanted to give (recommended textbook A or recommended textbook B). Then, the participants completed the power scale for the third time (Power 3, Cronbach's *α* = .91). In the end, they also answered several questions about their motivations. These questions were the same as those in Study 1, except that one question (i.e., “To what extent did you want to harm the questioner?”) was not involved.

### Results and discussion

#### Manipulation check

**FIGURE 2:**
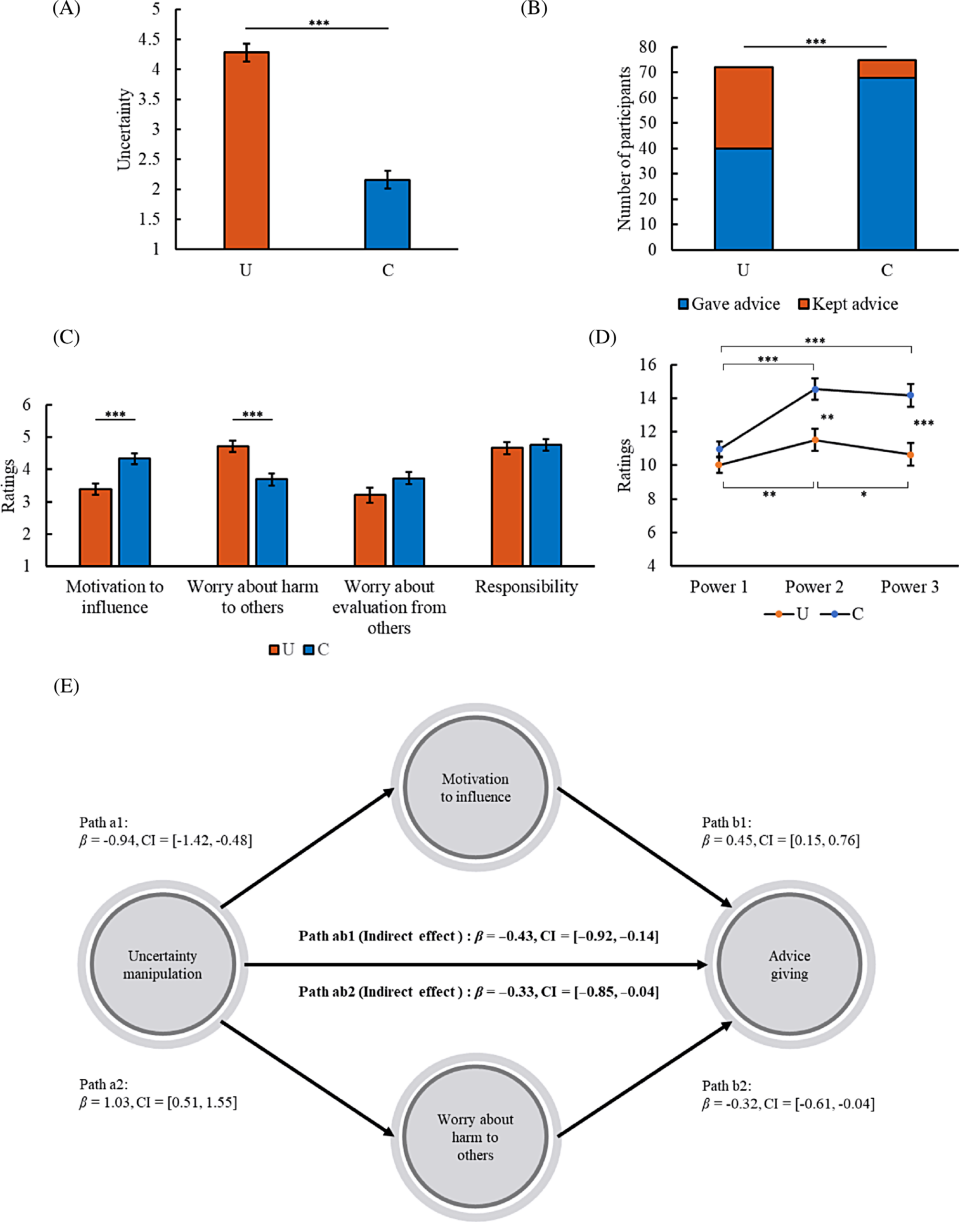
Results of Study 2. (A) Mean uncertainty ratings (± *SE*) in the uncertainty (U) and certainty (C) conditions. (B) Number of participants who gave advice to others or kept advice to themselves in the U and C conditions. (C) Mean ratings of motivations related to advice giving (± *SE*) in the U and C conditions. (D) Mean ratings of sense of power at different time points (± *SE*) in the U and C conditions. (E) Motivation to influence and worry about harm to others could mediate the effect of uncertainty on advice giving. *p < .05, **p < .01, ***p < .001. *β*, path coefficient; CI, 95% confidence interval of *β*; bold font, significant indirect effect.

The participants in the uncertainty condition were more uncertain about which book was better than those in the certainty condition (*F*(1,142) = 103.60, *p < *.001, partial *η* ^2^ = .422; Figure 2A).

#### Advice giving

The percentage of participants who gave advice or kept advice differed between the conditions (*χ* ^2^(1,147) = 23.23, *p < *.001, Cramer's *V* = .398; Figure 2B).

Among the given advice, most of the advice was consistent with the information from the imaginary scenario and the participants' own opinion (see SI 8 for the related results of Studies 2, 3, and 4), which implies that uncertainty does not change people's behavioral pattern of giving advice based on the information they have and their own opinions (e.g., Hadar & Fischer, 2008).

#### Motivations related to advice giving

The participants in the uncertainty condition were less motivated to influence others' choice (*F*(1,145) = 15.64, *p < *.001, partial *η* ^2^ = .097) and had more worry that their advice might cause harm to others' benefits (*F*(1,145) = 15.47, *p < *.001, partial *η* ^2^ = .096) than those in the certainty condition (Figure 2C). There was no significant difference in worry about evaluation from others or responsibility between the conditions (all *F* < 3.08, *p* > .081, partial *η* ^2^ < .021).

#### Sense of power

No significant difference was found in Power 1 between the conditions (*F*(1,145) = 1.68, *p = *.197, partial *η* ^2^ = .011). Compared to keeping advice, giving advice increased the sense of power after advice behavior (Power 3; *F*(1,145) = 10.99, *p = *.001, partial *η* ^2^ = .070).

The participants in the uncertainty condition had lower Power 2 (*F*(1,145) = 10.59, *p = *.001, partial *η* ^2^ = .068) and Power 3 (*F*(1,145) = 14.41, *p < *.001, partial *η* ^2^ = .090) than those in the certainty condition (Figure 2D).

In the uncertainty condition, Power 2 was higher than Power 1 (*F*(1,71) = 7.11, *p = *.009, partial *η* ^2^ = .091); Power 3 was lower than Power 2 (*F*(1,71) = 4.96, *p = *.029, partial *η* ^2^ = .065) but not different from Power 1 (*F*(1,71) = 1.14, *p = *.289, partial *η* ^2^ = .016). In the certainty condition, Power 2 was higher than Power 1 (*F*(1,74) = 27.78, *p < *.001, partial *η* ^2^ = .273); Power 3 was higher than Power 1 (*F*(1,74) = 22.67, *p < *.001, partial *η* ^2^ = .234) but not different from Power 2 (*F*(1,74) = .94, *p = *.336, partial *η* ^2^ = .013).

The findings demonstrate that the inhibitory effect of uncertainty on the sense of power emerges when advisors know the situation related to advice giving.

#### Mediation effect

The motivation to influence and worry about harm to others mediated the effect of uncertainty on advice giving (Table S7). Power 2 did not have a significant mediation effect. None of the other mediation effects reached significance.

To test whether motivation to influence and worry about harm to others were independently involved in the effect of uncertainty on advice giving, we put both motivation to influence and worry about harm to others into the model as mediators simultaneously. Their mediation effects remained significant (Figure 2E).

The uncertainty we manipulated in Studies 1 and 2 could be considered as a combination of ambiguity and risk. To disentangle them, we developed two novel interpersonal paradigms for examining the pure ambiguity effect (in Study 3) and pure risk effect (in Study 4) on advisors.

## STUDY 3

### Methods

#### Participants and design

One hundred and fifty college students participated in our experiment. Invalid responses from 32 were excluded (SI 3), leaving 118 participants in the analyses (100 females, 18 males, *M*_age_ = 21.85 years, *SD*_age_ = 3.11 years; 60 in the uncertainty condition, 58 in the certainty condition). Our study had a between‐subject design.

#### Procedure and measures

At the beginning, participants completed a power scale (Power 1, Cronbach's *α* = .71; Schaerer et al., 2018). Then, they took part in a novel game we developed, the development of which was inspired by Vives and Feldmanhall (2018). This game included an advisor and a decider. The decider needed to make decisions on six buckets. Each bucket contained 100 balls. Some balls were white, while the others were black. One ball would be randomly drawn from each bucket. The decider needed to guess the color of each drawn ball (i.e., six guesses in total). Each correct guess earned the decider 1 Chinese yuan, while each wrong guess earned 0. The decider had no information about the number of white or black balls in each bucket, but they could receive advice from the advisor before making guesses.

The advisor was informed of some of the information about the number of white and black balls in each bucket (e.g., black: 10, unknown color [could be black or white]: 85, white: 5). They chose whether to give advice to the decider after being aware of the information of each bucket. The advisor received 6 Chinese yuan as a participation fee regardless of their choice or performance in the game. This manipulation helped eliminate the possibility that the advisor was reluctant to help the decider to earn money by giving advice when the advisor got nothing (i.e., inequity aversion). Both the advisor and decider knew all the rules of this game.

All participants played the role of the advisor in the game. In addition to choosing whether to give advice, they needed to answer a series of questions. The participants were informed that only their choice about advice (i.e., “no advice,” “white was recommended,” or “black was recommended”) would be delivered to a decider (a stranger to them) in the future, but their answers to the other questions would not. During the game, we set cues to remind the participants whether their responses in the current part would be shown to the decider.

**FIGURE 3:**
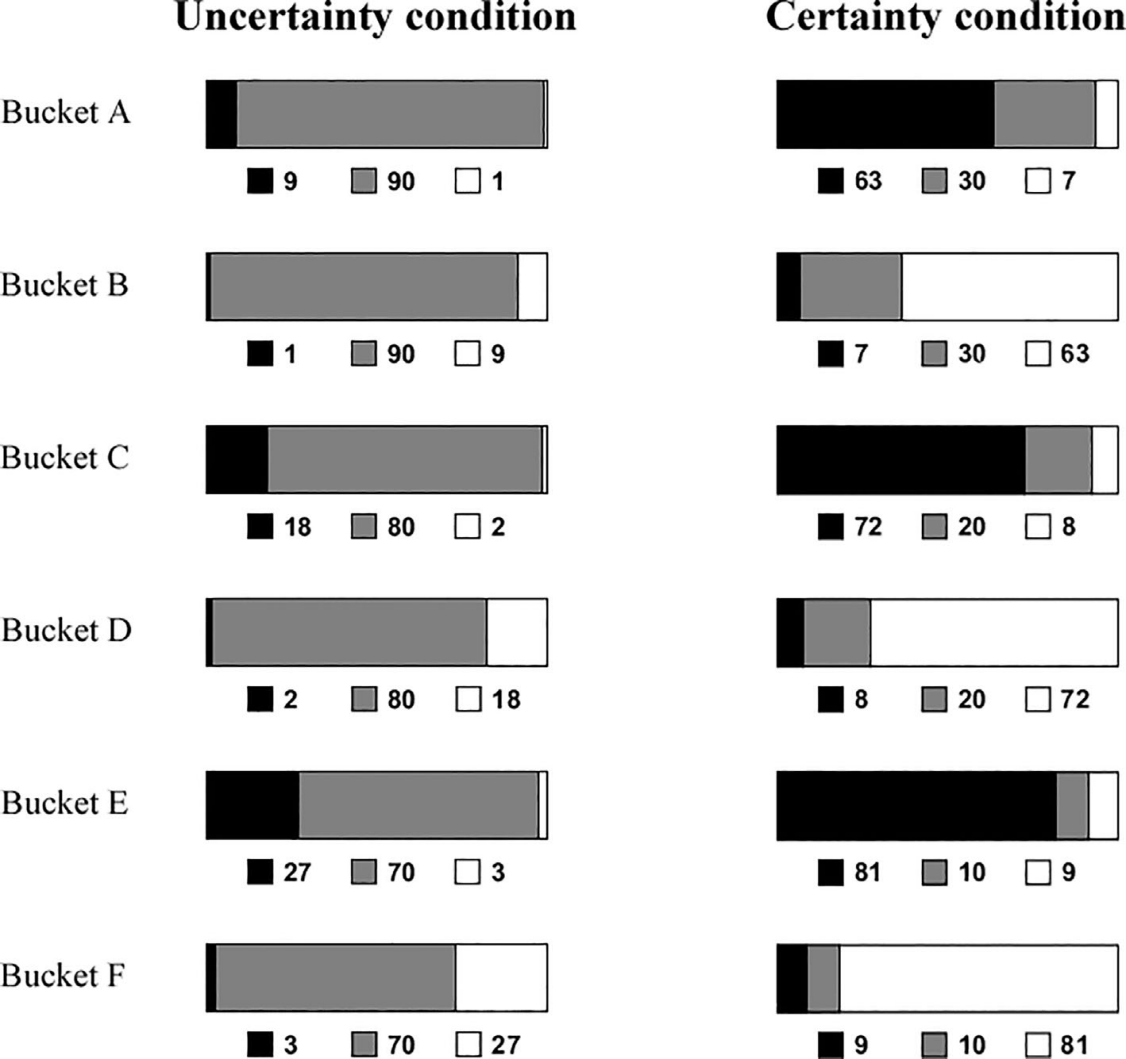
The information of each bucket presented to participants in the uncertainty and certainty conditions in Study 3. The numbers of black, white, and unknown (could be black or white) balls are indicated by black, white, and gray patches, respectively.

After a comprehension test (SI 7), the game started. The participants were given information about the buckets one by one. The numbers of black, white, and unknown balls are indicated by black, white, and gray patches, respectively. Based on the known information, the proportion of majority balls to minority balls was always 9:1 for all buckets. In the uncertainty condition, the number of unknown balls varied from 70 to 90. In the certainty condition, the number of unknown balls varied from 10 to 30 (Figure 3). Thus, we manipulated uncertainty by changing ambiguity (Vives & Feldmanhall, 2018). In both the uncertainty and certainty conditions, the information about buckets A and B, buckets C and D, and buckets E and F were the same, except that the color of the majority balls and minority balls was swapped. This manipulation attempted to eliminate the influence of participants' color preferences on our results. The buckets were presented in the following order: buckets F, B, C, A, E, and D. After seeing the information about each bucket, the participants speculated on the color of the randomly drawn ball (black or white) and indicated their certainty (1 = *completely uncertain*, 7 = *completely certain*). Then, the participants completed the power scale for the second time (Power 2, Cronbach's *α* = .87). Following the power scale, the participants saw each bucket once again and chose whether to give advice to the decider. If the participants chose to give advice on a bucket, they needed to indicate what advice they wanted to give (recommend black or recommend white). Afterwards, the participants completed the power scale for the third time (Power 3, Cronbach's *α* = .92). At the end, they also answered several questions about their motivations. These questions were the same as those in Study 1 except that the question “To what extent did you want to harm the questioner?” was replaced by “To what extent did you wanted to help the decider?” (motivation to help, one item; this helped us to know whether the influence participants wanted to exert was benign).

### Results and discussion

#### Manipulation check

**FIGURE 4:**
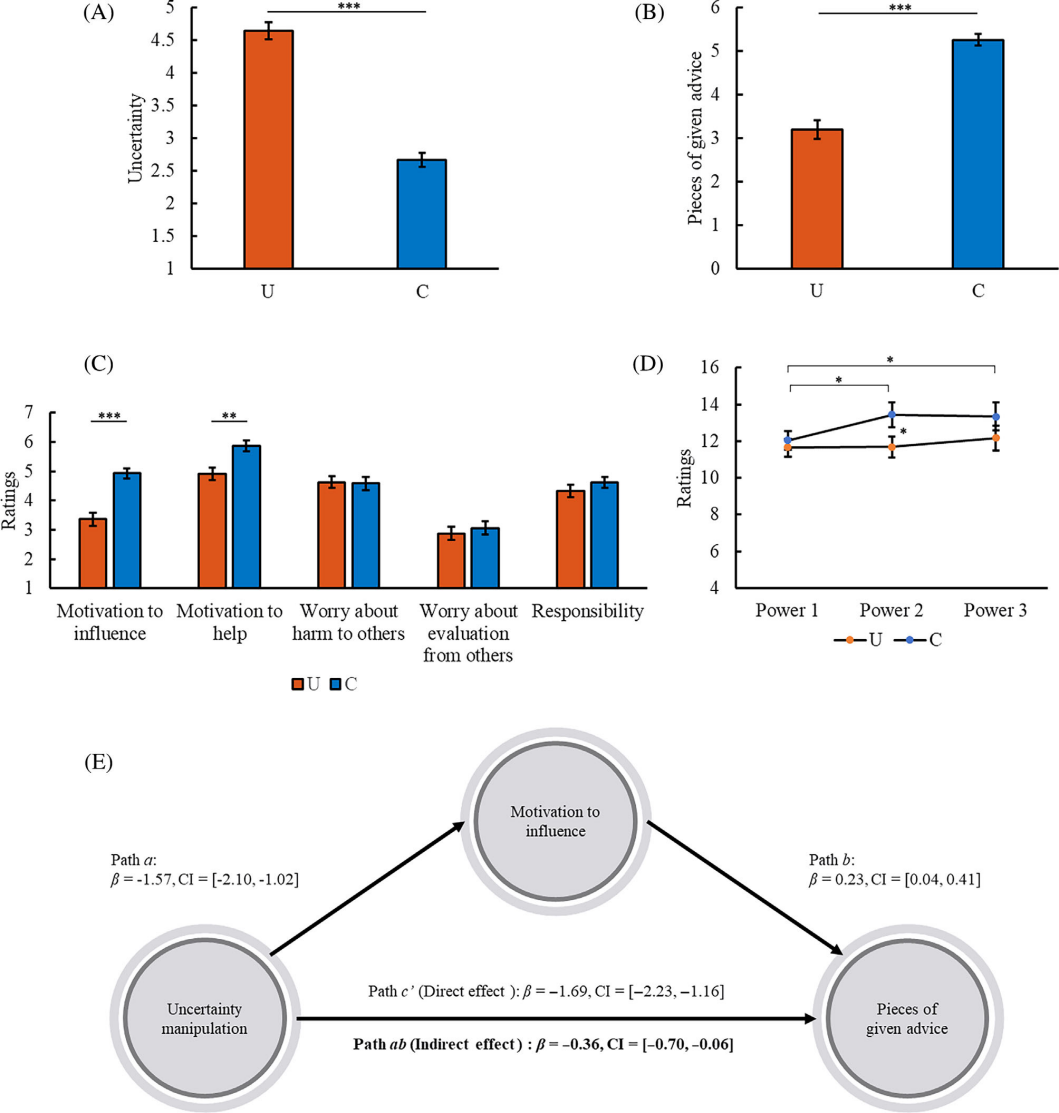
Results of Study 3. (A) Mean uncertainty ratings (± *SE*) in the uncertainty (U) and certainty (C) conditions. (B) Mean pieces of given advice (± *SE*) in the U and C conditions. (C) Mean ratings of motivations related to advice giving (± *SE*) in the U and C conditions. (D) Mean ratings of sense of power at different time points (± *SE*) in the U and C conditions. (E) Motivation to influence mediated the effect of uncertainty on pieces of given advice. *p < .05, **p < .01, ***p < .001. *β*, path coefficient; CI, 95% confidence interval of *β*; bold font, significant indirect effect.

On average, the participants in the uncertainty condition had higher uncertainty ratings than those in the certainty condition (*F*(1,116) = 131.89, *p < *.001, partial *η* ^2^ = .532; Figure 4A).

#### Advice giving

The participants in the uncertainty condition gave fewer pieces of advice than those in the certainty condition (*U* = 501.50, *Z* = 6.88, *p < *.001, *r* = .634; Figure 4B).

#### Motivations related to advice giving

The participants in the uncertainty condition were less motivated to influence the decider's choice (*F*(1,116) = 30.59, *p < *.001, partial *η* ^2^ = .209) and were less inclined to help the decider (*F*(1,116) = 11.35, *p = *.001, partial *η* ^2^ = .089) than those in the certainty condition (Figure 4C). There was no significant difference in worry about harm to others, worry about evaluation from others, or responsibility between the conditions (all *Fs* < 1.10, *ps* > .297, partial *η* ^2^ *s*< .009).

We found a significant correlation between the motivation to influence and motivation to help (Pearson correlation *r* = .53, *p < *.001, *N* = 118), which implies that the influence the participants wanted to exert on the decider was benign.

#### Sense of power

No significant difference was found in Power 1 between the conditions (*F*(1,116) = .33, *p = *.569, partial *η* ^2^ = .003). In line with previous findings (Schaerer et al., 2018), a linear regression revealed that pieces of advice were positively correlated with the sense of power after advice behavior (Power 3), although the effect was not significant (*β* = .36, *SE* = .30, *t* = 1.20, *p = *.235).

The participants in the uncertainty condition had lower Power 2 than those in the certainty condition (*F*(1,116) = 3.99, *p = *.048, partial *η* ^2^ = .033; Figure 4D). No significant difference was found in Power 3 between the conditions (*F*(1,116) = 1.36, *p = *.246, partial *η* ^2^ = .012).

In the uncertainty condition, Power 2 was not different from Power 1 (*F*(1,59) = .01, *p = *.942, partial *η* ^2^ < .001); Power 3 was not different from Power 1 (*F*(1,59) = .76, *p = *.386, partial *η* ^2^ = .013) or Power 2 (*F*(1,59) = 1.77, *p = *.188, partial *η* ^2^ = .029). In the certainty condition, Power 2 was higher than Power 1 (*F*(1,57) = 5.64, *p = *.021, partial *η* ^2^ = .090); and Power 3 was higher than Power 1 (*F*(1,57) = 4.17, *p = *.046, partial *η* ^2^ = .068) but not different from Power 2 (*F*(1,57) = .04, *p = *.842, partial *η* ^2^ = .001).

The findings support that ambiguity inhibits the sense of power when advisors know the situation related to advice giving.

#### Mediation effect

Motivation to influence (Figure 4E) and motivation to help (Table S8) mediated the effect of uncertainty on the number of given advice. Power 2 did not have a significant mediation effect. None of the other psychological factors had a significant mediation effect.

In the following Study 4, we examined the effect of pure risk on advisors.

## STUDY 4

### Methods

#### Participants and design

One hundred and forty‐six college students participated in our experiment. Invalid responses from 16 participants were excluded (SI 3), leaving 130 participants in the analyses (74 females, 56 males, *M*_age_ = 21.38 years, *SD*_age_ = 2.12 years; 66 in the uncertainty condition, 64 in the certainty condition). Our study had a between‐subject design.

#### Procedure and measures

**FIGURE 5:**
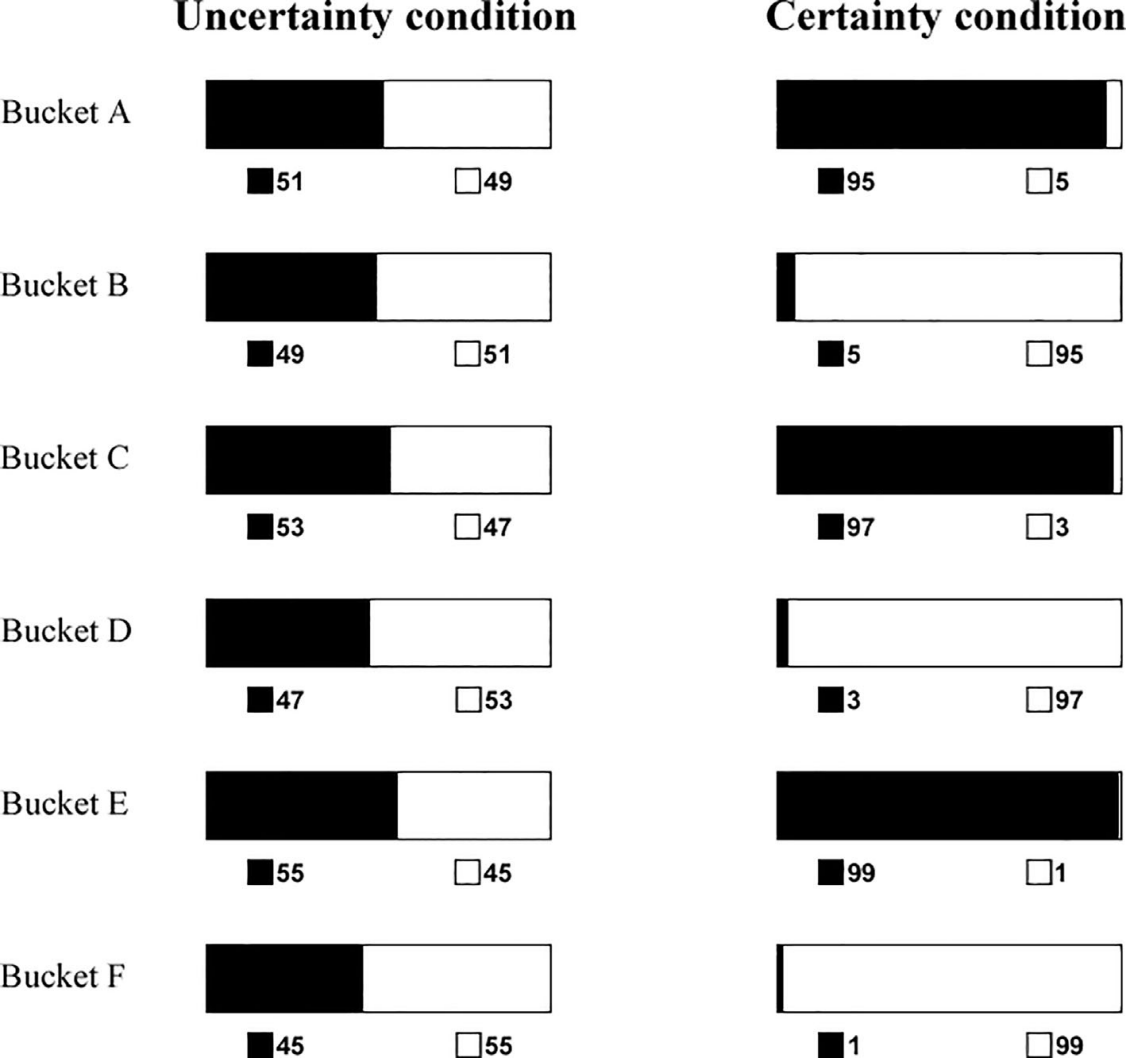
The information of each bucket presented to participants in the uncertainty and certainty conditions in Study 4. The numbers of black and white balls are indicated by black and white patches, respectively.

The procedure and measures of Study 4 were similar to Study 3. The difference was that the participants as advisors in Study 4 could obtain the complete information about the number of white and black balls in each bucket. Specifically, in the uncertainty condition, the proportion of majority balls to minority balls varied from 51:49 to 55:45 (Figure 5); in the certainty condition, it varied from 95:5 to 99:1. Thus, here we manipulated uncertainty by changing risk (other than ambiguity; Vives & Feldmanhall, 2018).

Like Study 3, the participants completed a power scale (Schaerer et al., 2018) at the beginning (Power 1, Cronbach's *α* = .88), after they obtained the information about the bucket (Power 2, Cronbach's *α* = .92), and after they completed the advice behavior (Power 3, Cronbach's *α* = .92).

### Results and discussion

#### Manipulation check

**FIGURE 6:**
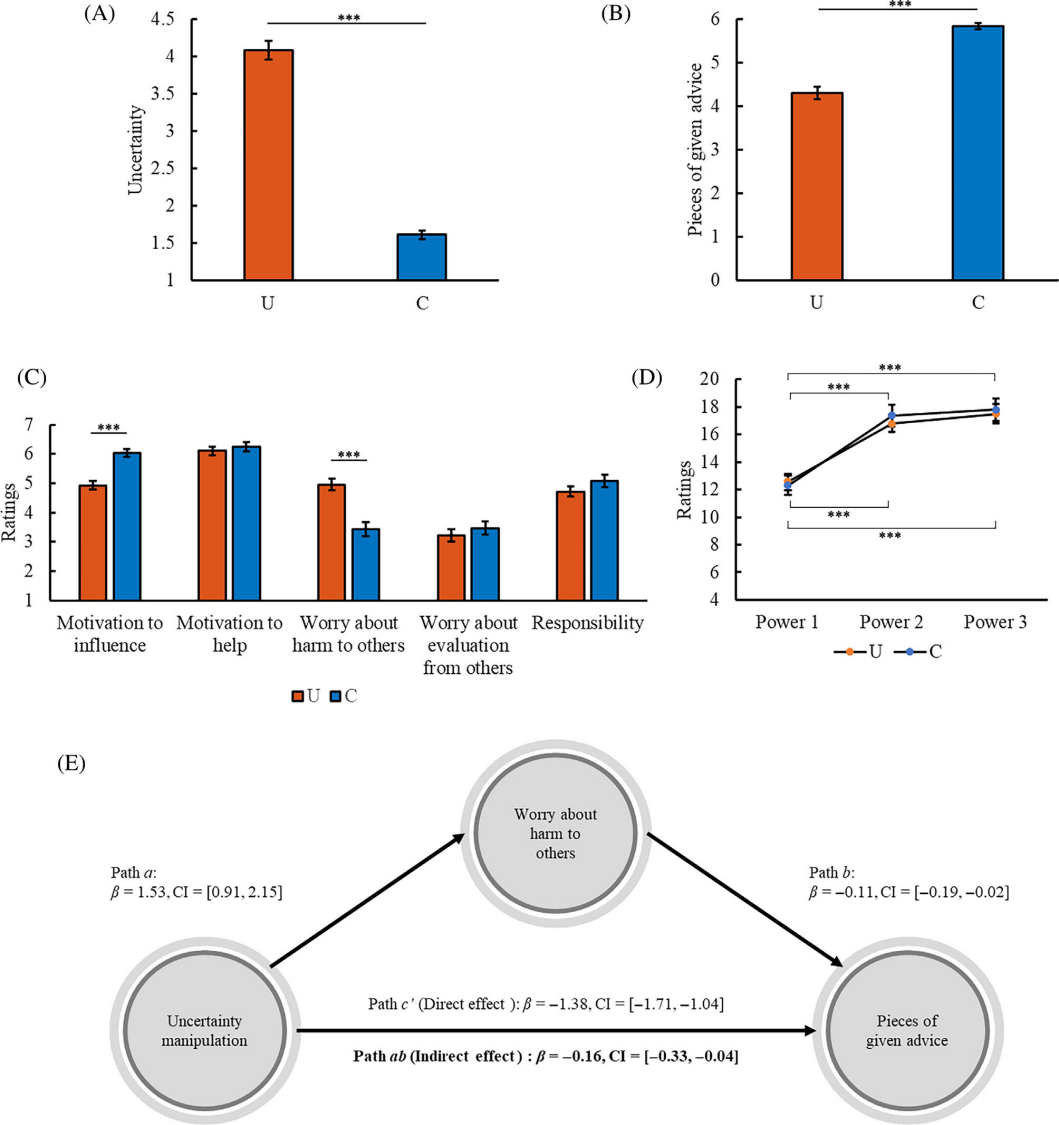
Results of Study 4. (A) Mean uncertainty ratings (± *SE*) in the uncertainty (U) and certainty (C) conditions. (B) Mean pieces of given advice (± *SE*) in the U and C conditions. (C) Mean ratings of motivations related to advice giving (± *SE*) in the U and C conditions. (D) Mean ratings of sense of power at different time points (± *SE*) in the U and C conditions. (E) Worry about harm to others mediated the effect of uncertainty on pieces of given advice. ***p < .001. *β*, path coefficient; CI, 95% confidence interval of *β*; bold font, significant indirect effect.

The participants in the uncertainty condition had higher uncertainty ratings than those in the certainty condition (*F*(1,128) = 313.99, *p < *.001, partial *η* ^2^ = .710; Figure 6A).

#### Advice giving

The participants in the uncertainty condition gave fewer pieces of advice than those in the certainty condition (*U* = 635.00, *Z* = 7.90, *p < *.001, *r* = .693; Figure 6B).

#### Motivations related to advice giving

The participants in the uncertainty condition were less motivated to influence the decider's choice (*F*(1,128) = 34.78, *p < *.001, partial *η* ^2^ = .214) and had more worry that their advice might cause harm to others' benefits (*F*(1,128) = 23.92, *p < *.001, partial *η* ^2^ = .157) than those in the certainty condition (Figure 6C). There was no significant difference in motivation to help, worry about evaluation from others, or responsibility between the two conditions (all *Fs* < 1.73, *ps* > .191, partial *η* ^2^ *s*< .013).

Again, we found a significant correlation between the motivation to influence and motivation to help (Pearson correlation *r* = .26, *p = *.003, *N* = 130).

#### Sense of power

No significant difference was found in Power 1 between the conditions (*F*(1,128) = .07, *p = *.800, partial *η* ^2^ = .001). There was a trend that the participants who gave more pieces of advice felt a stronger sense of power after advice behavior (Power 3; *β* = .78, *SE* = .45, *t* = 1.73, *p = *.087).

No significant difference was found in Power 2 (*F*(1,128) = .32, *p = *.574, partial *η* ^2^ = .002) or Power 3 (*F*(1,128) = .08, *p = *.782, partial *η* ^2^ = .001) between the conditions (Figure 6D).

In the uncertainty condition, Power 2 was higher than Power 1 (*F*(1,65) = 88.51, *p < *.001, partial *η* ^2^ = .577); Power 3 was higher than Power 1 (*F*(1,65) = 81.20, *p < *.001, partial *η* ^2^ = .555) but not different from Power 2 (*F*(1,65) = 3.82, *p = *.055, partial *η* ^2^ = .055). Similarly, in the certainty condition, Power 2 was higher than Power 1 (*F*(1,63) = 45.45, *p < *.001, partial *η* ^2^ = .419); and Power 3 was higher than Power 1 (*F*(1,63) = 49.06, *p < *.001, partial *η* ^2^ = .438) but not different from Power 2 (*F*(1,63) = 0.90, *p = *.346, partial *η* ^2^ = .014).

The findings demonstrate that risk does not affect the sense of power in the situation of advice giving.

#### Mediation effect

Worry about harm to others mediated the effect of uncertainty on the amount of given advice (Figure 6E). Motivation to influence did not have a significant mediation effect. None of the other psychological factors had a significant mediation effect (Table S9).

The effects of ambiguity and risk on advisors share some similarities, but also have differences. These are further discussed in the General Discussion section.

## GENERAL DISCUSSION

We developed novel paradigms to manipulate advisors' uncertainty and systematically studied their behavioral and psychological responses. In line with our hypothesis, we found that advisors under uncertainty give less advice. It provides insights into why advisor–advisee communication is common in daily life (Kassirer et al., 2020; Kramer, 2016; Parnell & Hammer, 2018). Previous research has suggested that advisees' trust in advisors decreases rapidly when advisees consistently receive inaccurate advice (Leong & Zaki, 2018). The observed negative association between uncertainty and advice giving indicates that advisors proactively monitor the quality of their advice in relation to the level of uncertainty, which is conducive to sustaining the efficiency of advisor–advisee communication.

In addition to the present novel finding that uncertainty inhibits advice giving, our study also uncovered the potential psychological mechanism of uncertainty‐modulated advice giving. The results of Studies 2, 3, and 4 reveal that uncertainty decreases advisors' motivation to influence. Furthermore, Studies 2 and 3 show that the motivation to influence mediates the effect of uncertainty on advice giving. These findings suggest that advisors who face uncertainty have reduced motivation to influence advisees' decisions, leading to a decrease in interpersonal influence attempts (i.e., advice giving). The observed decrease in motivation to influence can be attributed to the understanding that advisors who are uncertain about the outcomes of advisees' choices have little chances of achieving their intended goals by influencing advisees' behavior (Ove Hansson, 1996) and may perceive diminished effectiveness in their attempts to exert influence.

We found a significant effect of uncertainty on worry about harm to others in Studies 1, 2, and 4. Studies 2 and 4 show that worry about harm to others mediates the effect of uncertainty on advice giving. These findings support the notion that interpersonal concerns play a significant role in the relationship between uncertainty and advice giving, aligning with previous studies that highlight the influence of interpersonal concerns on advice giving (Barneron & Yaniv, 2020; Mahmoodi et al., 2018).

To avoid misunderstanding, we note that we adopted the mediation analyses to elaborate the correlational rather than causal relationships among the variables (Fiedler et al., 2011). These attempts, which help to identify the candidates of causal mediators, could be considered as initial steps for identifying causal mechanisms. Based on our findings, future studies could further examine whether the motivation to influence and worry about harm to others causally mediate the effect of uncertainty on advice giving using moderation‐of‐process designs (Imai et al., 2013).

Replicating previous findings (Schaerer et al., 2018), we found that advice giving increases advisors' sense of power (Power 3). The effects were significant in Studies 1 and 2 but insignificant in Studies 3 and 4.

In addition to the replication, we tested a new hypothesis that uncertainty inhibits the sense of power. It was examined at two time points (Power 2 and 3). The results of Power 2 suggest a unique contribution of uncertainty to the sense of power, regardless of the effects of the power baseline and advice giving (SI 9). It is noted that Power 2 was measured when the participants were aware of the advice‐related situation but before the advice behavior. The findings support that the effect of uncertainty on the sense of power emerges during the evaluation of the situation in the advisors' minds, which echoes recent theoretical perspectives that the sense of power derives from a subjective judgment about one's own ability to influence others (Tost, 2015).

The results revealed that the sense of power (Power 2) did not mediate the effect of uncertainty on advice giving. Taking into account Schaerer et al.'s (2018) findings that giving advice enhances the sense of power after the advice behavior, it is suggested that while advice giving may predict subsequent feelings of power, the sense of power prior to the advice behavior does not play a mechanistic role in uncertainty‐modulated advice behavior. Instead, the sense of power appears to emerge concurrently with the advice‐giving process.

The general pattern of the effects of uncertainty on Power 3 is similar to that on Power 2. It implies that apart from imagining, recalling, or experiencing a situation related to advice, imagining, recalling, or conducting advice behavior does not have an additional effect on the sense of power. These findings indicate that the influence of uncertainty on the sense of power is not contingent upon the specific behaviors associated with advice giving.

Studies 3 and 4, respectively, manipulated pure ambiguity and pure risk, which provides us a chance to compare their effects. We found two interesting differences. One is that ambiguity, but not risk, inhibits sense of power. Compared with risk, people are more averse to ambiguity (Camerer & Weber, 1992; Curley et al., 1986; Slovic & Tversky, 1974). Increased ambiguity, relative to increased risk, can induce stronger negative feelings, which more severely diminishes advisors' positive feelings (i.e., sense of power; e.g., Russell, 2003). Therefore, the effect size of ambiguity on sense of power is larger than that of risk. Larger sample size may be needed to observe a significant effect of risk. The other difference is that risk but not ambiguity increases worry about harm to others. Due to the lack of information of possibility, events in ambiguity are believed to be more unpredictable that those in risk (Hsu et al., 2005; Volz et al., 2004). This feature may make advisors under ambiguity experience a relatively high level of worry about harm to others, irrespective of the degree of ambiguity. It can explain why the participants' worry about harm to others remained stable despite the variations in ambiguity.

Some pioneering studies demonstrated the influences of uncertainty and uncertainty‐related preference (e.g., ambiguity tolerance) on helping behaviors (Kappes et al., 2018; Vives & Feldmanhall, 2018). However, the advice‐giving behavior that we are interested in is distinct from helping behaviors. The helping behaviors investigated by previous studies have direct effects on others' monetary gain, while the advice‐giving behavior we study only has indirect effects on others' benefits (i.e., it affects advisees only if the advisees decide to take the advice). We contend that the distinction between helping behavior and advice giving highlights the need to more thoroughly illustrate the role of uncertainty in advice giving and its underlying psychological mechanisms.

Several remaining open questions should be investigated in future studies. First, while our study found the mediation effects of the motivation to influence and worry about harm to others, it would be valuable to explore other potential mediators. For example, anticipated guilt (Chang et al., 2011; De Hooge et al., 2014) and anticipated pride (Mobbs et al., 2015) may play roles in the relationship between uncertainty and advice giving. Second, we focused on situations where advisors had no conflicts of interest with advisees' benefits. However, it is important to know that advisors sometimes face situations involving conflicts of interest (Barneron & Yaniv, 2020). Future studies should examine how uncertainty affects advisors' behavior in different situations, including those characterized by conflicting interests. Third, although we successfully manipulated uncertainty by changing the information‐related probability (ambiguity and risk), participants' feelings of uncertainty could also depend on how they construe their representation of the task (e.g., some may overestimate their ability to predict the order in which the balls will be drawn; Szollosi et al., 2022). Future studies could incorporate measures to assess participants' subjective representation and construal of uncertainty, and examine their roles in advice giving. Fourth, our Study 2 adopted an imagination paradigm. Social studies provided evidence of some differences in behavior between hypothetical and real choices (Camerer & Mobbs, 2017). The validity and credibility will be increased if the findings can be replicated in real situations by future studies. Fifth, one may notice that whereas uncertainty consistently showed a significant effect on advice giving in all four studies, its influences on motivations and sense of power varied across different studies. One possible reason is that the effect size of uncertainty on advice giving is larger. Thus, we could better identify its effect on advice giving regardless of the paradigms used and the types of uncertainty involved. It is encouraged to use a larger sample size to investigate the effects of uncertainty on motivations and emotions in the future. Sixth, our findings suggest an inhibitory effect of uncertainty on sense of power. Previous studies imply that people under uncertainty are more likely to believe in the power of social institutions and high‐status members as a strategy to obtain sense of power (Landau et al., 2015; Melamed et al., 2019). An interesting future direction is to examine which compensatory strategies are adopted by people for restoring the reduced sense of power caused by uncertainty and whether they are useful.

In conclusion, our studies decipher the psychological and behavioral responses of advisors in the face of uncertainty. We found that uncertainty inhibits advice giving and modulates the motivation to influence, worry about harm to others, and sense of power. The motivation to influence and worry about harm to others mediate the relationship between uncertainty and advice giving. Two distinct types of uncertainty, ambiguity and risk, have subtly different effects on advisors' psychological activities (i.e., sense of power and worry about harm to others). Our findings shed light on the relationship between uncertainty and advice giving, thereby contributing to a deeper understanding of advisor–advisee communication from the perspective of advisors.

## FUNDING INFORMATION

Ruida Zhu is supported by the National Natural Science Foundation of China (NSFC) (32200884) and the Start‐up Project for Support of Young Doctors (SL2023A04J00351). Chao Liu is supported by the National Natural Science Foundation of China (NSFC) (32271092, 32130045), the Major Project of National Social Science Foundation (19ZDA363), the Beijing Municipal Science and Technology Commission (Z151100003915122), and the National Program for Support of Top‐Notch Young Professionals. Song Su is supported by the National Natural Science Foundation of China (NSFC) (71872016).

## CONFLICT OF INTEREST STATEMENT

All authors declare that they have no conflicts of interest.

## ETHICS STATEMENT

The procedures used in the studies adhered to the standards set by the Declaration of Helsinki and were approved by local Research Ethics Committee at the State Key Laboratory of Cognitive Neuroscience and Learning, Beijing Normal University. Written informed consents were obtained from the participants.

## Supporting information


**Data S1:** Supporting Information.

## Data Availability

Data and materials and that support the findings are publicly available on an Open Science Framework (OSF) repository (https://osf.io/d9syq/?view_only=ce60581f55ab41f68cf08694b9b68f1a).
