# Supplementary material for: When advisors do not know what is best for advisees: Uncertainty inhibits advice giving
Source: Psych J. 2024 Mar 26;13(4):663–78. doi: 10.1002/pchj.745 (PMC11317185; doi:10.1002/pchj.745)
Supplement: Supplementary file 1 — Data S1: Supporting Information. [file PCHJ-13-663-s001.docx]

**Supplementary information**

**SI 1 Power analyses**

In Study 1, we expected a medium effect size of uncertainty on advice giving in a chi-square test. Using G*Power 3 software (Faul et al., 2007), we set the expected effect size (*w* = 0.30), probability of type I error (0.05), power (1 - *β* = 0.80), and degree of freedom (*df* = 1) and determined the minimum sample size to be 88 participants. Our sample (134 valid participants) had 80% power to detect effects of *w* > 0.24 at *p* < 0.05 (2-tailed tests).

In Study 2, consistent with Study 1, we determined the minimum sample size to be 88 participants. Our sample (147 valid participants) had 80% power to detect effects of *w* > 0.23 at *p* < 0.05 (2-tailed tests).

In Study 3, we expected a medium effect size of uncertainty on pieces of advice in a Mann-Whitney test. Using G*Power 3 software (Faul et al., 2007), we set the expected effect size (*d* = 0.60), probability of type I error (0.05), power (1 - *β* = 0.80), and distribution (no assumption) and determined the minimum sample size to be 104 participants. Our sample (118 valid participants) had 80% power to detect effects of *d* > 0.56 at *p* < 0.05 (2-tailed tests).

In Study 4, consistent with Study 3, we determined the minimum sample size to be 104 participants. Our sample (130 valid participants) had 80% power to detect effects of *d* > 0.53 at *p* < 0.05 (2-tailed tests).

**SI 2 Description of missing data**

In Study 1, two participants did not give certainty ratings; six participants did not rate the clearness of their advice; nine participants did not report the occurrence time of the recalled events; two participants did not give certainty ratings and did not rate the clearness of their advice; three participants did not give certainty ratings and did not report the occurrence time of recalled events; one participants did not give certainty ratings, did not rate the clearness of their advice, and did not report the occurrence time of the recalled event; one participant did not report the occurrence time of the recalled event and their age; three participants did not answer a filler question when they filled in the power scale; two participants did not report the occurrence time of the recalled events and did not answer a filler question when they filled in the power scale.

In Study 2, one participant did not give certainty rating; two participants did not give certainty ratings and did not report their opinions about which textbook was better; two participants did not answer a filler question when they filled in the power scale; two participants did not report what advice they wanted to give, though they chose to give advice.

In Study 3, one participant did not answer a filler question when they filled in the power scale; one participant did not report what advice they wanted to give, though they chose to give advice.

We assigned missing values to the unanswered questions and still involved the other data of these participants in the analyses as they did not violate the exclusion rules.

**SI 3 Excluded participants**

**Table S1.** The number of excluded participants according to different rules.

| Exclusion rules | Study 1 | Study 2 | Study 3 | Study 4 |
| --- | --- | --- | --- | --- |
| Did not pass an attention check | 4 | 7 | 3 | 1 |
| Did not answer more than five questions | 5 | 3 | 1 | N/A^[[1]](#footnote-1)^ |
| Did not answer the key questions (i.e., questions about advice giving or sense of power) | 5 | 1 | 0 | N/A^1^ |
| Did not pass a comprehension test | N/A^[[2]](#footnote-2)^ | N/A^2^ | 28 | 15 |

**SI 4 Attention check**

| This question is for checking whether you pay an attention to our experiment. Please choose number 6 to indicate that you are carefully reading and answering our questions.  1 - 2 - 3 - 4 - 5 - 6 - 7 |
| --- |

**SI 5 Effect of uncertainty on the sense of power**

In Study 1, when the ratings of Power 1 and advice giving (0 = kept advice, 1 = gave advice) were involved in the ANCOVAs as covariates, the differences between the conditions in the ratings of Power 2 (uncertainty vs. certainty condition: *F*(1,130) = 4.64, *p* = 0.033, partial *η*^2^ = 0.034; Power 1: *F*(1,130) = 78.15, *p* < 0.001, partial *η*^2^ = 0.375; advice giving: *F*(1,130) = 0.02, *p* = 0.880, partial *η*^2^ < 0.001) and Power 3 (uncertainty vs. certainty condition: *F*(1,130) = 4.99, *p* = 0.027, partial *η*^2^ = 0.037; Power 1: *F*(1,130) = 60.78, *p* < 0.001, partial *η*^2^ = 0.319; advice giving: *F*(1,130) = 0.31, *p* = 0.577, partial *η*^2^ = 0.002) remained significant. We also examined the effect of uncertainty on sense of power when uncertainty was indexed by the participants’ uncertainty ratings instead of the conditions (uncertainty vs. certainty). We replicated our previous findings. A linear regression showed a significant effect of uncertainty ratings on Power 2 (*β* = -0.68, *SE* = 0.30, *t* = 2.27, *p* = 0.025), even when the effects of Power 1 (*β* = 0.82, *SE* = 0.10, *t* = 8.43, *p* < 0.001) and advice giving (*β* = 0.58, *SE* = 0.79, *t* = 0.74, *p* = 0.464) were controlled (Table S2). Another linear regression showed that uncertainty ratings were negatively correlated with Power 3, but the effect was not significant (*β* = -0.27, *SE* = 0.32, *t* = 0.83, *p* = 0.408) when the effects of Power 1 (*β* = 0.79, *SE* = 0.10, *t* = 7.65, *p* < 0.001) and advice giving (*β* = 1.62, *SE* = 0.84, *t* = 1.91, *p* = 0.058) were controlled.

In Study 2, when the ratings of Power 1 and advice giving (0 = kept advice, 1 = gave advice) were involved in the ANCOVAs as covariates, the differences between the conditions in ratings of Power 2 (uncertainty vs. certainty condition: *F*(1,143) = 6.59, *p* = 0.011, partial *η*^2^ = 0.044; Power 1: *F*(1,143) = 33.58, *p* < 0.001, partial *η*^2^ = 0.190; advice giving: *F*(1,143) = 0.17, *p* = 0.685, partial *η*^2^ = 0.001) and Power 3 (uncertainty vs. certainty condition: *F*(1,143) = 7.55, *p* = 0.007, partial *η*^2^ = 0.050; Power 1: *F*(1,143) = 31.23, *p* < 0.001, partial *η*^2^ = 0.179; advice giving: *F*(1,143) = 1.98, *p* = 0.162, partial *η*^2^ = 0.014) remained significant. A linear regression showed a significant effect of uncertainty ratings on Power 2 (*β* = -0.91, *SE* = 0.30, *t* = 3.08, *p* = 0.003), even when the effects of Power 1 (*β* = 0.60, *SE* = 0.10, *t* = 6.00, *p* < 0.001) and advice giving (*β* = -0.13, *SE* = 1.10, *t* = 0.12, *p* = 0.905) were controlled (Table S3). Another linear regression showed a significant effect of uncertainty ratings on Power 3 (*β* = -0.94, *SE* = 0.30, *t* = 3.17, *p* = 0.002), even when the effects of Power 1 (*β* = 0.57, *SE* = 0.10, *t* = 5.68, *p* < 0.001) and advice giving (*β* = 0.92, *SE* = 1.10, *t* = 0.84, *p* = 0.403) were controlled.

In Study 3, when the ratings of Power 1 and pieces of advice were involved in the ANCOVAs as covariates, no significant difference was found in Power 2 (uncertainty vs. certainty condition: *F*(1,114) = 1.35, *p* = 0.248, partial *η*^2^ = 0.012; Power 1: *F*(1,114) = 60.53, *p* < 0.001, partial *η*^2^ = 0.347; advice giving: *F*(1,114) = 0.56, *p* = 0.458, partial *η*^2^ = 0.005) or Power 3 (uncertainty vs. certainty condition: *F*(1,114) = 0.05, *p* = 0.819, partial *η*^2^ < 0.001; Power 1: *F*(1,114) = 47.36, *p* < 0.001, partial *η*^2^ = 0.294; advice giving: *F*(1,114) = 0.89, *p* = 0.347, partial *η*^2^ = 0.008) between the conditions. A linear regression showed a significant effect of uncertainty ratings on Power 2 (*β* = -1.25, *SE* = 0.31, *t* = 4.03, *p* < 0.001), even when the effects of Power 1 (*β* = 0.72, *SE* = 0.09, *t* = 8.06, *p* < 0.001) and pieces of advice (*β* = -0.19, *SE* = 0.25, *t* = 0.78, *p* = 0.435) were controlled (Table S4). Another linear regression showed a significant effect of uncertainty ratings on Power 3 (*β* = -1.30, *SE* = 0.37, *t* = 3.51, *p* = 0.001), even when the effects of Power 1 (*β* = 0.75, *SE* = 0.11, *t* = 7.01, *p* < 0.001) and pieces of advice (*β* = -0.26, *SE* = 0.30, *t* = 0.89, *p* = 0.375) were controlled.

In Study 4, when the ratings of Power 1 and pieces of advice were involved in the ANCOVAs as covariates, no significant difference was found in Power 2 (uncertainty vs. certainty condition: *F*(1,126) = 0.54, *p* = 0.462, partial *η*^2^ = 0.004; Power 1: *F*(1,126) = 69.10, *p* < 0.001, partial *η*^2^ = 0.354; advice giving: *F*(1,126) = 0.01, *p* = 0.914, partial *η*^2^ < 0.001) or Power 3 (uncertainty vs. certainty condition: *F*(1,126) = 0.50, *p* = 0.482, partial *η*^2^ = 0.004; Power 1: *F*(1,126) = 52.98, *p* < 0.001, partial *η*^2^ = 0.296; advice giving: *F*(1,126) = 2.70, *p* = 0.103, partial *η*^2^ = 0.021) between the conditions. A linear regression showed no significant effect of uncertainty ratings on Power 2 (*β* = -0.48, *SE* = 0.38, *t* = 1.25, *p* = 0.213), when the effects of Power 1 (*β* = 0.65, *SE* = 0.08, *t* = 8.15, *p* < 0.001) and pieces of advice (*β* = -0.22, *SE* = 0.47, *t* = 0.47, *p* = 0.637) were controlled (Table S5). Another linear regression showed no significant effect of uncertainty ratings on Power 3 (*β* = 0.10, *SE* = 0.42, *t* = 0.23, *p* = 0.816), when the effects of Power 1 (*β* = 0.64, *SE* = 0.09, *t* = 7.34, *p* < 0.001) and pieces of advice (*β* = 0.68, *SE* = 0.52, *t* = 1.31, *p* = 0.192) were controlled.

**SI 6 Serial multiple mediation**

According to the advice of an anonymous reviewer, we created a more complex model with two mediators. Specifically, we adopted a serial multiple mediation analysis to examine whether an indirect effect of uncertainty on advice giving is achieved through the worry about harm to others and motivation to influence in sequence (i.e., uncertainty → worry about harm to others → motivation to influence → advice giving). The indirect effect of uncertainty on advice giving through worry about harm to others tendency and harm avoidance in sequence was not significant in Study 1 (*β* = 0.0006, 95% CI = [-0.02, 0.03]). It was not significant in Studies 2 (*β* = 0.0159, 95% CI = [-0.06, 0.11]), 3 (*β* = 0.0003, 95% CI = [-0.02, 0.02]), or 4 (*β* = -0.0008, 95% CI = [-0.03, 0.02]), either.

**SI 7 Comprehension test (for Study 3)**

| **Which role will you play in the game?**  1) Advisor  2) Decider |
| --- |
| **Each bucket contains 100 balls. The balls are either black or white.**  1) The statements are correct.  2) The statements are wrong. |
| **The figure below indicates that _______**  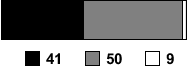  1) The bucket contains 9 black, 41 white, and 50 unknown colour balls.  2) The bucket contains 41 black, 9 white, and 50 unknown colour balls.  3) The bucket contains 50 black, 9 white, and 41 unknown colour balls. |
| **Which statement is correct?**  1) The advisor can choose to give advice to the decider or not.  2) The advisor must give advice to the decider. |
| **Which statement is correct?**  1) Each correct guess earns the decider 1 yuan  2) The decider earns 6 yuan regardless of their choice or performance in the game. |

**SI 8 Consistency of advice giving**

In Study 2, among the given advice, most of them were consistent with the information from the imaginary scenario^[[3]](#footnote-3)^ (i.e., recommending the textbook A) (uncertainty condition: consistent, 92.50%, inconsistent, 7.50%; certainty condition: consistent, 95.45%, inconsistent, 4.55%). There was no significant difference in the consistency between the given advice and the information from the scenario between the two conditions (Fisher’s exact test, *N* = 106, *p* = 0.670, Cramer’s *V* = 0.062). Among the given advice, most of them were in line with the participants’ own opinion (uncertainty condition: consistent, 97.44%, inconsistent, 2.56%; certainty condition: consistent, 100%, inconsistent, 0%). There was no significant difference in the consistency between the given advice and the participants’ own opinions between the two conditions (Fisher’s exact test, *N* = 104, *p* = 0.375, Cramer’s *V* = 0.127).

In Study 3, among the given advice most of them were consistent with the known information^[[4]](#footnote-4)^ (uncertainty condition: *M* = 94.33%, *SD* = 20.10%, *Mdn* = 100%; certainty condition: *M* = 95.95%, *SD* = 15.73%, *Mdn* = 100%). There was no significant difference in the consistency between the given advice and the known information between the two conditions (*U* = 1504.00, *Z* = 0.05, *p* = 0.963, *r* = 0.005). Among the given advice most of them were consistent with the participants’ own speculations (uncertainty condition: *M* = 97.05%, *SD* = 9.71%; *Mdn* = 100%; certainty condition: *M* = 98.02%, *SD* = 7.05%; *Mdn* = 100%). There was no significant difference in the consistency between the given advice and the participants’ own speculations between the two conditions (*U* = 1486.50, *Z* = 0.26, *p* = 0.796, *r* = 0.025).

In Study 4, among the given advice most of them were consistent with the information^[[5]](#footnote-5)^ (uncertainty condition: *M* = 99.23%, *SD* = 4.35%, *Mdn* = 100%; certainty condition: *M* = 98.57%, *SD* = 9.57%, *Mdn* = 100%). There was no significant difference in the consistency between the given advice and the information between the two conditions (*U* = 2079.00, *Z* = 0.02, *p* = 0.987, *r* = 0.002). Among the given advice most of them were consistent with the participants’ own speculations (uncertainty condition: *M* = 99.23%, *SD* = 4.35%, *Mdn* = 100%; certainty condition: *M* = 98.57%, *SD* = 9.57%, *Mdn* = 100%). There was no significant difference in the consistency between the given advice and the participants’ own speculations between the two conditions (*U* = 2079.00, *Z* = 0.02, *p* = 0.987, *r* = 0.002).

**SI 9 Results of regression analyses**

**Table S2**. Effects of the uncertainty ratings, Power 1, and advice giving on the sense of power (Power 2 or Power 3) in Study 1.

| Independent variables | Power 2 | Power 3 |
| --- | --- | --- |
| Uncertainty ratings | -0.68^*^ (0.30) | -0.27 (0.32) |
| Power 1 | 0.82^***^ (0.10) | 0.79^***^ (0.10) |
| Advice giving | 0.58 (0.79) | 1.62 (0.84) |
| Intercept | 4.53^*^ (1.88) | 2.25 (2.01) |
| Observations | 126 | 126 |
| *R*^2^ | 0.43 | 0.37 |
| *F* statistic | 30.33^***^ | 24.10^***^ |

Note. Linear regression coefficients and standard errors (in parentheses) are reported. ^*^*p* < 0.05, ^***^*p* < 0.001.

**Table S3**. Effects of the uncertainty ratings, Power 1, and advice giving on the sense of power (Power 2 or Power 3) in Study 2.

| Independent variables | Power 2 | Power 3 |
| --- | --- | --- |
| Uncertainty ratings | -0.91^**^ (0.30) | -0.94^**^ (0.30) |
| Power 1 | 0.60^***^ (0.10) | 0.57^***^ (0.10) |
| Advice giving | -0.13 (1.10) | 0.92 (1.10) |
| Intercept | 9.80^***^ (1.79) | 8.81^***^ (1.78) |
| Observations | 144 | 144 |
| *R*^2^ | 0.27 | 0.28 |
| *F* statistic | 16.98^***^ | 18.24^***^ |

Note. Linear regression coefficients and standard errors (in parentheses) are reported. ^**^*p* < 0.01, ^***^*p* < 0.001.

**Table S4**. Effects of the uncertainty ratings, Power 1, and pieces of advice on the sense of power (Power 2 or Power 3) in Study 3.

| Independent variables | Power 2 | Power 3 |
| --- | --- | --- |
| Uncertainty ratings | -1.25^***^ (0.31) | -1.30^**^ (0.37) |
| Power 1 | 0.72^***^ (0.09) | 0.75^***^ (0.11) |
| Pieces of advice | -0.19 (0.25) | -0.26 (0.30) |
| Intercept | 9.40^***^ (2.29) | 9.74^**^ (2.75) |
| Observations | 118 | 118 |
| *R*^2^ | 0.44 | 0.37 |
| *F* statistic | 30.02^***^ | 22.47^***^ |

Note. Linear regression coefficients and standard errors (in parentheses) are reported. ^**^*p* < 0.01, ^***^*p* < 0.001.

**Table S5**. Effects of the uncertainty ratings, Power 1, and pieces of advice on the sense of power (Power 2 or Power 3) in Study 4.

| Independent variables | Power 2 | Power 3 |
| --- | --- | --- |
| Uncertainty ratings | -0.48 (0.38) | 0.10 (0.42) |
| Power 1 | 0.65^***^ (0.08) | 0.64^***^ (0.09) |
| Pieces of advice | -0.22 (0.47) | 0.68 (0.52) |
| Intercept | 11.54^***^ (3.44) | 6.00 (3.77) |
| Observations | 130 | 130 |
| *R*^2^ | 0.36 | 0.32 |
| *F* statistic | 23.92^***^ | 19.44^***^ |

Note. Linear regression coefficients and standard errors (in parentheses) are reported. ^***^*p* < 0.001.

**SI 10 Results of mediation analyses**

**Table S6.** Results of the mediation effects of each of six psychological measures on the relationship between uncertainty and advice giving in Study 1.

| Mediators | Path a | Path b | Path c’  (Direct effect) | Path ab  (Indirect effect) |
| --- | --- | --- | --- | --- |
| Motivation to influence | -0.21  [-0.67, 0.25] | 0.03  [-0.26, 0.32] | -2.29  [-3.21, -1.37] | -0.01  [-0.13, 0.07] |
| Motivation to harm | -0.17  [-0.54, 0.17] | -0.17  [-0.68, 0.26] | -2.34  [-3.27, -1.40] | 0.02  [-0.07, 0.15] |
| Worry about harm to others | 0.62  [0.14, 1.11] | -0.13  [-0.46, 0.15] | -2.23  [-3.16, -1.31] | -0.08  [-0.37, 0.08] |
| Worry about evaluation from others | -0.04  [-0.59, 0.51] | -0.06  [-0.34, 0.22] | -2.30  [-3.23, -1.38] | 0.003  [-0.09, 0.10] |
| Responsibility | 0.33  [-0.16, 0.82] | -0.24  [-0.64, 0.12] | -2.27  [-3.19, -1.35] | -0.07  [-0.29, 0.08] |
| Power 2 | -2.61  [-4.28, -0.94] | 0.01  [-0.09, 0.12] | -2.27  [-3.21, -1.33] | -0.03  [-0.31, 0.26] |

Note: Path a, from the predictor variable to the mediator variable; Path b, from the mediator variable to the outcome variable. The coefficient of the path and its 95% confidence interval was reported.

**Table S7.** Results of the mediation effects of each of five psychological measures on the relationship between uncertainty and advice giving in Study 2.

| Mediators | Path a | Path b | Path c’  (Direct effect) | Path ab  (Indirect effect) |
| --- | --- | --- | --- | --- |
| Motivation to influence | -0.94  [-1.41, -0.48] | 0.43  [0.14, 0.73] | -1.78  [-2.72, -0.85] | **-0.39**  **[-0.82, -0.11]** |
| Worry about harm to others | 1.03  [0.50, 1.54] | -0.28  [-0.62, -0.01] | -1.84  [-2.77, -0.91] | **-0.27**  **[-0.72, -0.01]** |
| Worry about evaluation from others | -0.52  [-1.10, 0.06] | 0.09  [-0.14, 0.34] | -2.01  [-2.92, -1.10] | -0.05  [-0.23, 0.09] |
| Responsibility | -0.10  [-0.60, 0.43] | -0.03  [-0.31, 0.24] | -2.05  [-2.96, -1.15] | 0.002  [-0.08, 0.10] |
| Power 2 | -3.01  [-4.85, -1.21] | 0.05  [-0.03, 0.13] | -1.94  [-2.86, -1.02] | -0.13  [-0.47, 0.09] |

Note: Path a, from the predictor variable to the mediator variable; Path b, from the mediator variable to the outcome variable. The coefficient of the path and its 95% confidence interval was reported. Bold font, significant indirect effect.

**Table S8.** Results of the mediation effects of each of six psychological measures on the relationship between uncertainty and advice giving in Study 3.

| Mediators | Path a | Path b | Path c’  (Direct effect) | Path ab  (Indirect effect) |
| --- | --- | --- | --- | --- |
| Motivation to influence | -1.57  [-2.10, -1.02] | 0.23  [0.04, 0.41] | -1.69  [-2.23, -1.16] | **-0.36**  **[-0.70, -0.06]** |
| Motivation to help | -0.94  [-1.47, -0.41] | 0.19  [0.02, 0.36] | -1.88  [-2.39, -1.37] | **-0.18**  **[-0.39, -0.02]** |
| Worry about harm to others | 0.05  [-0.54, 0.66] | -0.12  [-0.26, 0.02] | -2.05  [-2.54, -1.56] | -0.01  [-0.09, 0.10] |
| Worry about evaluation from others | -0.18  [-0.78, 0.42] | 0.03  [-0.12, 0.17] | -2.05  [-2.55, -1.56] | -0.01  [-0.07, 0.05] |
| Responsibility | -0.29  [-0.82, 0.25] | -0.09  [-0.27, 0.10] | -2.08  [-2.58, -1.59] | 0.02  [-0.04, 0.14] |
| Power 2 | -1.75  [-3.50, -0.06] | 0.01  [-0.04, 0.06] | -2.04  [-2.54, -1.54] | -0.02  [-0.14, 0.08] |

Note: Path a, from the predictor variable to the mediator variable; Path b, from the mediator variable to the outcome variable. The coefficient of the path and its 95% confidence interval was reported. Bold font, significant indirect effect.

**Table S9.** Results of the mediation effects of each of six psychological measures on the relationship between uncertainty and advice giving in Study 4.

| Mediators | Path a | Path b | Path c’  (Direct effect) | Path ab  (Indirect effect) |
| --- | --- | --- | --- | --- |
| Motivation to influence | -1.11  [-1.48, -0.74] | 0.11  [-0.04, 0.25] | -1.42  [-1.77, -1.07] | -0.12  [-0.31, 0.05] |
| Motivation to help | -0.14  [-0.55, 0.26] | 0.07  [-0.06, 0.20] | -1.53  [-1.84, -1.22] | -0.01  [-0.05, 0.03] |
| Worry about harm to others | 1.53  [0.91, 2.15] | -0.11  [-0.19, -0.02] | -1.38  [-1.71, -1.04] | **-0.16**  **[-0.33, -0.04]** |
| Worry about evaluation from others | -0.24  [-0.86, 0.38] | 0.06  [-0.03, 0.15] | -1.53  [-1.84, -1.22] | -0.01  [-0.07, 0.03] |
| Responsibility | -0.37  [-0.92, 0.18] | -0.01  [-0.11, 0.09] | -1.54  [-1.86, -1.23] | 0.003  [-0.04, 0.06] |
| Power 2 | -0.57  [-2.58, 1.43] | 0.01  [-0.02, 0.04] | -1.54  [-1.85, -1.22] | -0.005  [-0.04, 0.03] |

Note: Path a, from the predictor variable to the mediator variable; Path b, from the mediator variable to the outcome variable. The coefficient of the path and its 95% confidence interval was reported. Bold font, significant indirect effect.

1. In Study 4, participants complete the experiment on computers. They could not submit their responses if they did not answer all the questions. In Studies 1, 2, and 3, instructions and stimuli were presented on paper. The participants responded to the questions using a pen and might miss some questions. [↑](#footnote-ref-1)
2. There was no comprehension test in Studies 1 and 2. [↑](#footnote-ref-2)
3. In both the uncertainty and control conditions, the description of the imaginary scenario implied that the textbook A was better. If participants recommended the textbook A in their advice, we considered their advice to be consistent with the information. [↑](#footnote-ref-3)
4. Regardless of the percentage of unknown balls, if the participants recommended the color of the majority balls based on what they knew, we considered the advice to be consistent with the information. [↑](#footnote-ref-4)
5. If the participants recommended the color of the majority balls, we considered the advice to be consistent with the information. [↑](#footnote-ref-5)
